# Laboratory considerations in the assessment of 25-hydroxyvitamin D in pregnant women by automated immunoassays

**DOI:** 10.1016/j.plabm.2024.e00430

**Published:** 2024-09-25

**Authors:** Darya Ayad Khalid, Bijan Nouri, Shakhawan Abdulrahman Mustafa, Mohammad Abdi

**Affiliations:** a Student Research Committee, Kurdistan University of Medical Sciences, Sanandaj, Iran; b Department of Epidemiology and Biostatistics, Faculty of Medicine, Kurdistan University of Medical Sciences, Sanandaj, Iran; c Kurdistan Institution for Strategic Studies and Scientific Research (KISSR), Sulaymaniyah, Kurdistan Region, Iraq; d Cellular and Molecular Research Center, Research Institute for Health Development, Kurdistan University of Medical Sciences, Sanandaj, Iran; e Department of Clinical Biochemistry, Faculty of Medicine, Kurdistan University of Medical Sciences, Sanandaj, Iran

**Keywords:** 25-Hydroxyvitamin D, Analytical method, Immunoassay, Inconsistency, Pregnant women

## Abstract

**Background:**

Because of the pathophysiological role of vitamin D in health, there is an increased interest to check the clinical status of this vitamin. Immunochemical assays are commonly employed to determine 25-hydroxyvitamin D (25 (OH) D) in clinical laboratories and its testing could be influenced by pre-analytic and analytic issues. The aim of this study was to compare the 25(OH)D results obtained from three commonly used immunoassays in pregnant women to check a possible discrepancy between tests.

**Material and methods:**

A group of 50 pregnant women who were in their third trimester were included in this study. The quantification of serum vitamin D was performed utilizing three immunochemistry-based assays including Elecsys, VIDAS and Alegria. We also involved 21 non-pregnant volunteers to clinically assess the vitamin D status in this group of people.

**Results:**

Our findings revealed a significant inconsistency between the obtained results from three assays for serum 25(OH)D. The 25(OH)D showed higher values when measured by the Elecsys assay while the VIDAS assay had lower values compared to the other immunoassays. More notably, the 25(OH)D testing in non-pregnant subjects showed consistent results in all three immunoassays.

**Conclusions:**

The results of the 25(OH)D measurements in pregnant women should be interpreted carefully due to a great inaccuracy in immunoassay testing. There is no such disagreement in non-pregnant people. Standardization of vitamin D testing in various settings is a crucial matter for clinical laboratories.

## Highlights

• We analyzed the possible inconsistency which may raise during vitamin D testing by immunochemical methods.
• We explored the analytical errors in three different immunoassays in pregnant women.
• Despite a large discrepancy between results of immunoassays in pregnant women, agreement was seen in non-pregnant females.

## Graphical abstract

**Figure:**
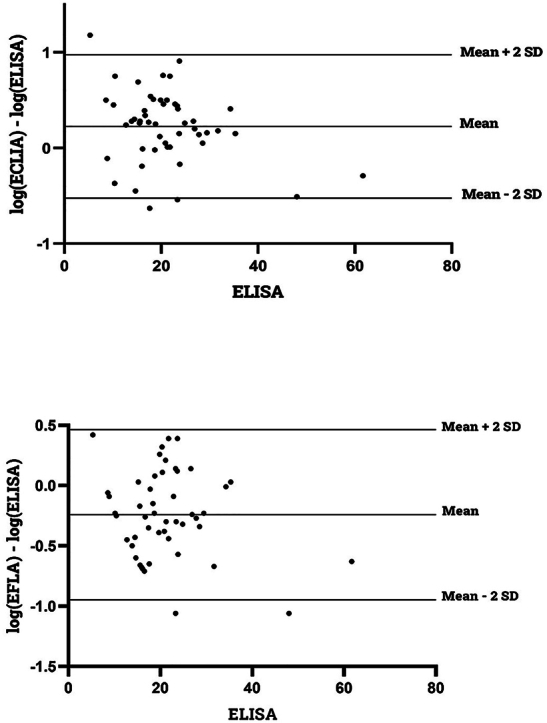

## 1 Introduction

Among all vitamin D metabolites, measuring 25-hydroxyvitamin D (25(OH)D) is a general agreement between clinical laboratories to assess the overall vitamin D status [1,2]. The serum concentration of 25(OH)D above 30 ng/ml (75 nmol/L) is recommended as the preferred level for this analyte [3]. However, levels higher than 100 ng/ml (250 nmol/L) are considered as hypervitaminosis D [4]. Similar target value (serum 25(OH)D > 30 ng/ml) has been approved by the Endocrine Society for vitamin D in pregnant women [5].

The detection methods for 25(OH)D can be broadly categorized into several distinct techniques [6]. Physical detection method like HPLC has been used for a long time as a preferred method for measuring 25(OH)D. Although no gold standard method has been introduced yet, but the LC-MS/MS technique is recently considered as the reference method for vitamin D assessment [7]. Immunoassay-based techniques are currently being utilized in almost all medical laboratories to evaluate clinical status of vitamin D [6,7] still, a number of pre-analytical and analytical factors should be considered regarding measurement of 25(OH)D by this method. Among all vitamin D metabolites, 24,25-dihydroxy vitamin D and especially 3-epi-25(OH)D3 showed significant cross reactivity with 25(OH)D in immunochemical based assays [[6], [7], [8]]. The main factor which determines the specificity of the test is the affinity of antibodies that are being used in such techniques [[6], [7], [8]]. Moreover, approximately 85–90 percent of vitamin D binds to vitamin D binding protein (VDBP), 10 to 15 percent forms a loose bond with albumin and only 1 % of this analyte presents in its free form in circulation. Therefore, incomplete separation from VDBP, particularly in automatic analyzers leads to the inaccurate measurement of this analyte. Various methods, including liquid-liquid extraction and solid-phase extraction, have been widely employed for this purpose [7].

There are also some pre-analytical issues that may affect vitamin D assessment in clinical laboratories. Among them, pregnancy is a specific situation that leads to a great alteration in hormones secretion which consequently causes a cascade of changes of proteins during pregnancy. It has been proved that the concentration of VDBP increases during pregnancy [9]. Approximately, 90 % of circulating 25(OH)D is attached to VDBP and releasing this protein-bonded fraction is the most pivotal step in clinical measurement of vitamin D [9]. It has been proved that estradiol is the main factor that increases VDBP in pregnant women [10].

Despite the above-mentioned influences, Immunoassay is the common technique for measuring 25(OH)D levels in clinical laboratories. Studies have revealed that serum level of VDBP may influence the measurement of 25(OH)D resulted from these methods. Heijboer A and colleagues [11] evaluated six assays utilized for measuring 25(OH)D and found that high concentration of VDBP in pregnant women interferes with vitamin D assay. Among five automated immunoassays, four assays showed a deviation from LC MS/MS results including Architect, Centaur, iSYS and Liaison and only Elecsys results was consistent with reference test.

In Iran, three fully automated assays are currently being used to clinically measure vitamin D status including Elecsys, VIDAS and Alegria. There is no standardized guideline for using immunoassay tests in clinical laboratories regarding vitamin D measurement in pregnant women. Therefore, we designed this study to evaluate the performance of the above mentioned three fully automated assays in the measurement of vitamin D levels in a group of pregnant women.

## 2 Materials and methods

### 2.1 Sample collection

Serum specimens were obtained from excess materials. Venous blood samples were collected from 50 pregnant women (20–45 years old) who were in the third trimester of their pregnancy, as indicated by their health records. All participants were referred to the community health centers for regular prenatal screening. Comprehensive anthropometric and clinical data, such as maternal and gestational age, intake of supplements and medication, and presence of comorbidities were extracted from medical records. After collection, samples were stored at −80 °C for further analysis. To evaluate the possible role of pregnancy on the tests results we also obtained 21 non-pregnant age-matched samples from volunteers (aged between 20 and 50 years). A signed informed consent was obtained from all individuals and the Ethics Committee of the Kurdistan University of Medical Sciences has approved all stages of this study.

### 2.2 Immunoassay testing of 25(OH)D

Within one month of collection, all samples were analyzed using commonly used immunoassay tests. Three immunoassays used in the present study were Elecsys Vitamin D total III (Roche Diagnostics), VIDAS® 25 OH Vitamin D TOTAL (Biomerieux, France) and 25-OH Vitamin D (Alegria). All assays use a competitive method which follow electrochemiluminescence, enzyme-linked fluorescent and enzyme-linked immunosorbent assays to measure 25(OH)D, respectively. The intraassay precision for the utilized assays were 6 %, 7 % and 10 % for Elecsys, VIDAS and Alegria, respectively.

### 2.3 Statistical analysis

Data analysis was conducted using the SPSS 16.0 and GraphPad Prism 8.2.1 statistical software. The results were presented as mean ± standard deviation. To examine the differences between two or multiple groups, *t*-test or one-way ANOVA analysis were employed, respectively. Furthermore, linear regression models were utilized to determine the correlation between two variables. The Bland-Altman analysis was used to determine the bias of results obtained from different assays. We also determined a possible agreement between three 25(OH)D assays through the Cohen's Kappa coefficient. A p-value below 0.05 was considered as statistically significant. All experiments were replicated three times.

## 3 Results

The average age (± standard deviation) of the subjects was 30(±6.9) years. All participants were in the third trimester of pregnancy and the mean age of gestation was 38 Weeks. The control samples were obtained from 21 non-pregnant females, whose ages ranged from 20 to 50 years.

**Fig. 1:**
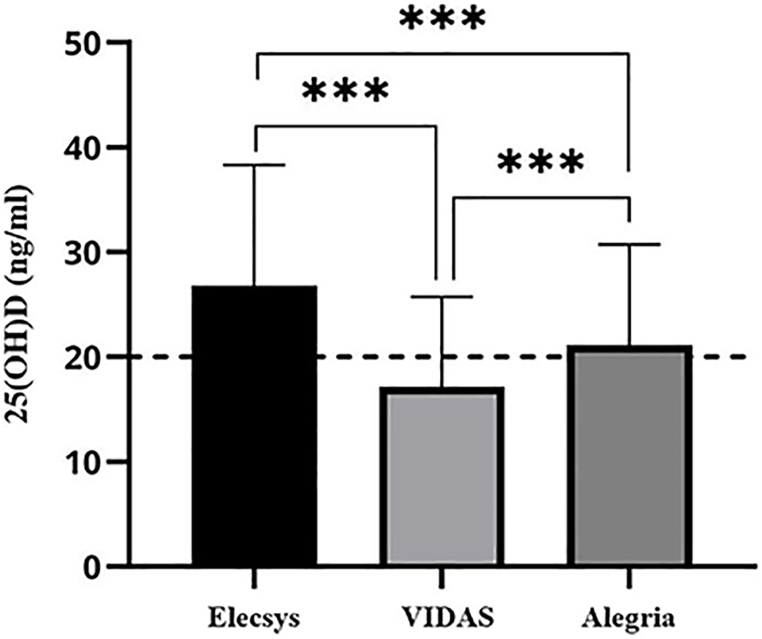
The concentration of 25(OH)D determined using three different methods. Serum 25(OH)D level (in ng/mL unit) had the highest values (26.8 ± 11.49 ng/mL) when measured by the Elecsys and decreasing concentration to 21.13 ± 9.59 and 17.13 ± 7.75 ng/mL for the Alegria and the VIDAS assays with a significant p value lower than 0.0001 for their difference.

The mean concentration of 25(OH)D in the studied subjects, as illustrated in Fig. 1, was 26.8, 17.13, and 21.13 ng/mL for the Elecsys, VIDAS and Alegria assays, respectively. Our findings confirmed that the difference observed in the 25(OH)D levels was statistically significant and can be attributed to the inconsistency between the Elecsys and VIDAS assays, the Elecsys and Alegria assays, and the VIDAS and Alegria assays (p value < 0.0001) (Fig. 1).

**Fig. 2:**
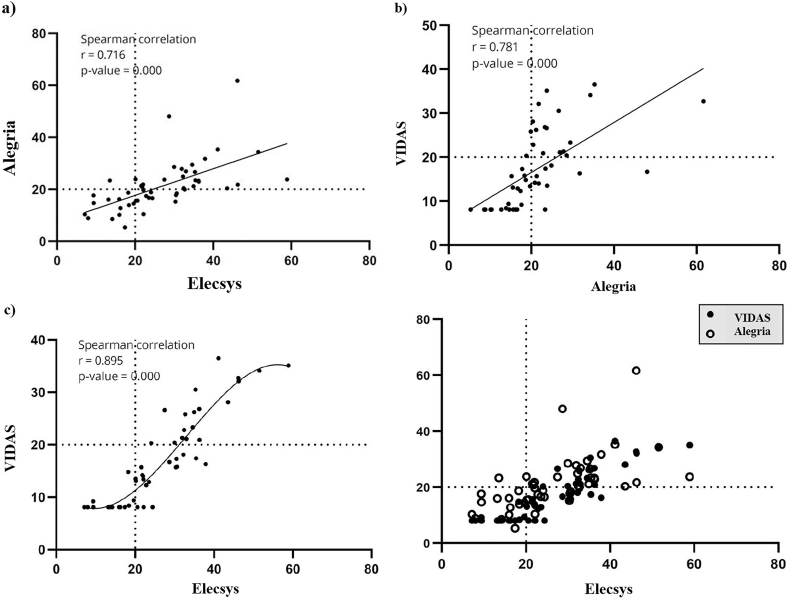
The correlation of the 25(OH)D results obtained from various assays: a) There was a significant correlation between the results obtained from the Elecsys and the Alegria assays, b) the VIDAS results had a direct statistically significant correlation with the Alegria results in the studied subject, c) the Elecsys results had also a statistically significant direct correlation with the results obtained from the VIDAS assay, and d) between all assays.

The obtained results from the Elecsys and Alegria assays showed a significant direct correlation between the results of the 25(OH)D obtained from these methods (Spearman's ρ correlation = 0.716, p value < 0.0001) (Fig. 2A). A similar significant correlation was also observed between the VIDAS and Alegria results (Spearman's ρ correlation = 0.781, p value < 0.0001) (Fig. 2B). Our results also revealed that the strongest correlation between the results of 25(OH)D measurement by the three immunoassays exists between the Elecsys and VIDAS assays (Spearman's ρ correlation = 0.895, p value < 0.0001) (Fig. 2C). Moreover, we analyzed a possible cumulative correlation between the obtained 25(OH)D results for the three assays and again, we found a significant positive correlation (Fig. 2D).

**Fig. 3:**
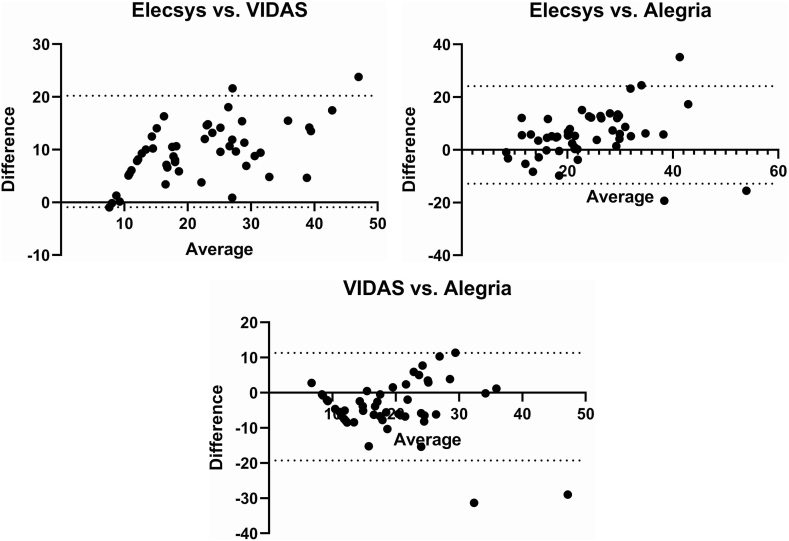
Comparison of 25(OH)D levels (ng/mL) results obtained from different immunoassays using the Bland-Altman difference plot: A) 25(OH)D concentrations obtained by Elecsys were generally higher (mean difference: +9.646 ng/mL) than those of VIDAS. B) Lower level of bias was also demonstrated for Elecsys compared to the Alegria results (mean bias: +5.671 ng/mL). C) On the contrary, the results obtained by VIDAS, were on average lower (mean bias: −3.975 ng/mL) than the Alegria.

We further analyzed the consistency between the results obtained from the different assays to find out if there is a clinically significant difference between them. According to the Bland-Altman analysis, the bias value between Elecsys and VIDAS assays was 9.646 ± 5.389 (Fig. 3A). The corresponding values for Elecsys vs. Alegria and VIDAS vs. Alegria assays were 5.671 ± 9.43 and −3.975 ± 7.811, respectively (Fig. 3B-C). Our results also showed that the Elecsys results were found to be between 8 % below and 4 % above the Alegria results. Besides, the VIDAS results were between 6 % below and 0 % above the Alegria results. Moreover, the Cohen's Kappa Index for the Elecsys and VIDAS assays was 0.359 (p value = 0.0005). The Kappa index for the Elecsys vs. Alegria and the VIDAS vs. Alegria assays were 0.480 (p value = 0.0001) and 0.560 (p value < 0.0001), respectively. These findings indicated a moderate agreement between the Elecsys and the Alegria assays and also between the VIDAS and the Alegria assays, while the Elecsys and the VIDAS assays were in fair agreement [12]. Accordingly, our finding showed that the difference between the results is so large that is unacceptable for clinical purposes.

Altogether, our findings showed that the results of the Elecsys assay had the highest values except for eleven samples (22 %) and in contrary, the results of the VIDAS assay showed the lowest values for all samples, except for 12 specimens (24 %).

**Fig. 4:**
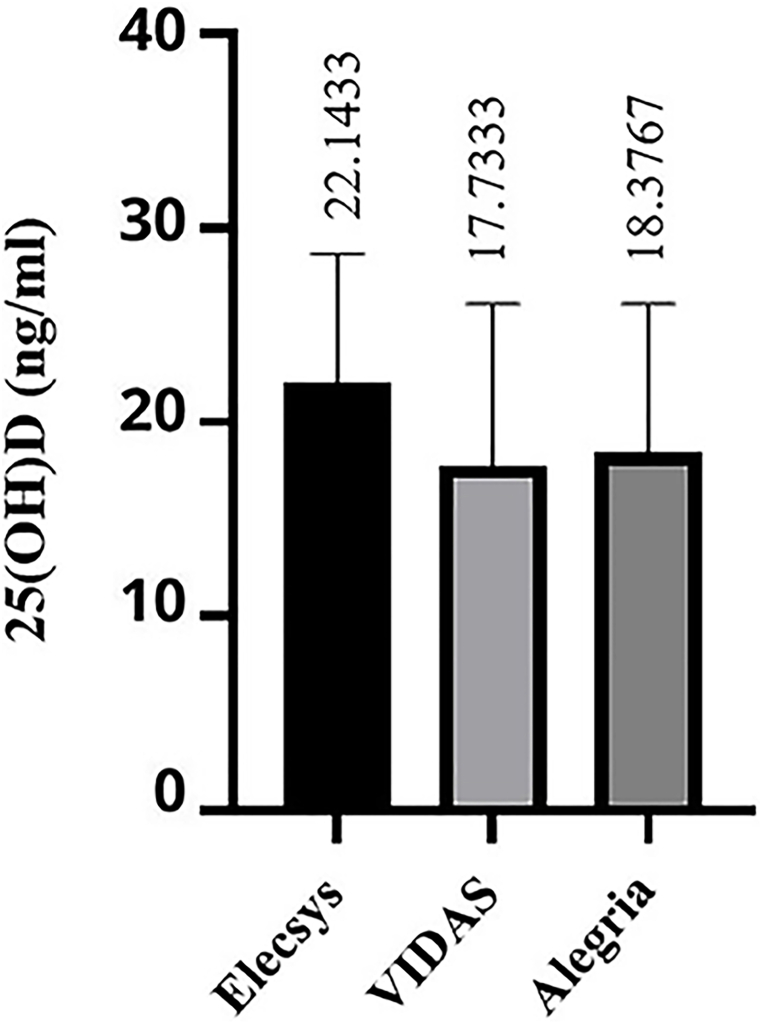
Serum 25(OH)D concentration in different assays: the 25(OH)D level (in ng/mL unit) starting with the Elecsys assay (22.14 ± 6.53 ng/mL) and decreasing down 18.38 ± 7.69 ng/mL for the Alegria followed by similar results obtained by the VIDAS assay (17.73 ± 8.35 ng/mL) with non-acceptable significance (p value = 0.065). According to the error plot, concentration of 25(OH)D in non-pregnant volunteers showed an agreement between results obtained from the three immunoassay tests.

More interestingly, the findings of our study revealed that among non-pregnant volunteers, the results of 25(OH)D measurement by the three fully automated immunoassays did not display any statistically significant difference (p = 0.65) (Fig. 4).

## 4 Discussion

In the present study, we compared the 25(OH)D results of three immunoassay tests in a group of pregnant women. Our finding revealed that there is no agreement between the obtained results from the Elecsys, VIDAS and Alegria assays and this discrepancy can highly increase the risk of misinterpretation of vitamin D status in this specific group. This misclassification leads to serious clinical consequences as vitamin D has an important role for health and is essential to support bone growth, particularly for infants. On the other hand, we showed that the results of 25(OH)D measurement by the Elecsys, VIDAS and Alegria assays were in accordance with each other when those assays utilized for non-pregnant age-matched people.

Inconsistency between immunoassay tests has been already reported in previous studies. Gallo S and colleagues [13] studied the analytical considerations in evaluation of 25(OH)D in infants. The vitamin D levels of capillary blood samples were measured by Octeia (EIA) and DiaSorin (RIA) assays. They also used LC MS/MS to evaluate various metabolites of vitamin D. They found that both EIA and RIA assays had poor accordance with LC MS/MS results and more importantly, they showed that EIA has greater cross-reactivity with 24,25(OH)2D3 than 3-epi-25(OH)D3. This group advised to measure 25(OH)D with LC MS/MS in early newborns (4–6 weeks of age). In another study by Heijboer A et al. [11], the performance of common immunoassay tests used for measuring 25(OH)D was assessed. They measured plasma/serum concentration of 25(OH)D in four groups including pregnant women, hemodialysis patients, intensive care patients and healthy individuals using five automated (Architect, Centaur, iSYS, Liaison, and Elecsys) and 1 RIA (Diasorin) assays. They also used LC MS/MS to confirm the results of immunoassays and ELISA to measure VDBP concentration in collected samples. Again, here they found a significant inaccuracy between immunoassay and LC MS/MS results. They proposed that the observed discrepancy is due to the concentration of VDBP. Accordingly, high concentration of VDBP inversely underestimate the 25(OH)D measurement.

Vitamin D and its metabolites are transferred in blood circulation mainly by VDBP. Only 1 % of vitamin D exists as free form in blood stream [14,15]. As a results, VDBP has a pivotal role in determining vitamin D status in clinical laboratories and releasing vitamin D from VDBP is one of the main steps in this process [6]. Discrepancy between the 25(OH)D results obtained from various immunoassay methods may partly derive from the methods and reagents used to detach vitamin D from its binding protein. Additionally, alteration in VDBP concentration in some pathologic and physiologic situations may lead to increase the errors of the measurement [11]. It has been clearly proved that estradiol significantly increases the hepatic production of VDBP, particularly in the third trimester of gestation [3,9,10,16]. Because of the importance of vitamin D in this period of pregnancy for proper growth of infant, inaccurate measurement of vitamin D status may lead to unwanted and undesirable clinical errors [17]. Therefore, standardization of method for evaluating vitamin D status is essential for clinical laboratories [18,19]. In line with previous studies, our findings revealed poor disagreement between 25(OH)D results obtained from three routine assays.

Our study has two limitations; first, because of technical limitation and inaccessibility to proper device, we were not able to confirm our results with the LC MS/MS reference method. However, a number of previous studies proved a great agreement between the results of the Elecsys and the LC MS/MS assays for 25(OH)D [11,20,21]. Besides, we used the latest generation of the Elecsys assay in our study which resolve most technical issues in the previous versions. Therefore, we assumed that the results of this assay may be closer to the clinical value. Second, we did not measure VDBP in our study, but the interfering role of VDBP on the measurement of 25(OH)D has been already revealed [11]. Also, it has been proved that the levels of VDBP reach to its higher concentration in the third trimester of gestation [9].

## 5 Conclusions

In summary, our study has an important conclusion; all approved assay for determining vitamin D status cannot be utilized in all groups of people. Particularly in pregnant women, standardization of method is essential. More importantly, in general situation economical methods like ELISA could be substituted instead of expensive methods like ECLIA and ELFA to clinically measure vitamin D status in low-income country.

## Disclosure of potential conflicts of interest

Mrs. D.A Khalid declares no potential conflicts of interest with respect to the research, authorship, and/or publication of this article. Dr. B Nouri declares that he has no conflict of interest. Dr. S. A. Mustafa declares that he has no conflict of interest. Dr. M Abdi has received research grants from the Kurdistan University of medical sciences.

## Ethical approval

All procedures performed in studies involving human participants followed the ethical standards of the ethics committee of Kurdistan University of Medical Sciences and with the 1964 Helsinki declaration and its later amendments or comparable ethical standards.

## Informed consent

Informed consent was obtained from all individual participants included in the study.

## Funding source

This work was supported by a research grant from the Kurdistan University of medical sciences (Grant/Award Numbers: 'IR.MUK.REC.1401.419′).

## Financial disclosure

The author has no financial relationships relevant to this article to disclose.

## Data availability statement

The data supporting this study's findings are available from the corresponding author upon reasonable request.

## CRediT authorship contribution statement

**Darya Ayad Khalid:** Formal analysis, Investigation, Writing – original draft. **Bijan Nouri:** Formal analysis, Methodology, Software, Writing – original draft, Writing – review & editing. **Shakhawan Abdulrahman Mustafa:** Conceptualization, Formal analysis, Methodology, Supervision, Writing – original draft, Writing – review & editing. **Mohammad Abdi:** Conceptualization, Data curation, Funding acquisition, Methodology, Project administration, Supervision, Validation, Writing – original draft, Writing – review & editing.

## Declaration of competing interest

The authors declare that they have no known competing financial interests or personal relationships that could have appeared to influence the work reported in this paper.

## Data Availability

Data will be made available on request.
